# Smoking among Young Rural to Urban Migrant Women in China: A Cross-Sectional Survey

**DOI:** 10.1371/journal.pone.0023028

**Published:** 2011-08-04

**Authors:** Xia Wan, Sanghyuk S. Shin, Qian Wang, H. Fisher Raymond, Huilin Liu, Ding Ding, Gonghuan Yang, Thomas E. Novotny

**Affiliations:** 1 Institute of Basic Medical Sciences of Chinese Academy of Medical Sciences, School of Basic Medicine of Peking Union Medical College, Beijing, China; 2 Joint Doctoral Program in Public Health, San Diego State University/University of California San Diego, San Diego, California, United States of America; 3 San Francisco Department of Public Health, San Francisco, California, United States of America; 4 Chinese Center for Disease Control and Prevention, Beijing, China; 5 Graduate School of Public Health, San Diego State University, San Diego, California, United States of America; Institute for Clinical Effectiveness and Health Policy (IECS), Argentina

## Abstract

**Background:**

Rural-to-urban migrant women may be vulnerable to smoking initiation as they are newly exposed to risk factors in the urban environment. We sought to identify correlates of smoking among rural-to-urban migrant women in China.

**Methods/Principal Findings:**

A cross-sectional survey of rural-to-urban migrant women working in restaurants and hotels (RHW) and those working as commercial sex workers (CSW) was conducted in ten provincial capital cities in China. Multiple logistic regression was conducted to identify correlates of smoking. We enrolled 2229 rural-to-urban migrant women (1697 RHWs aged 18–24 years and 532 CSWs aged 18–30 years). Of these, 18.4% RHWs and 58.3% CSWs reported ever tried smoking and 3.2% RHWs and 41.9% CSWs reported current smoking. Participants who first tried smoking after moving to the city were more likely to be current smokers compared to participants who first tried smoking before moving to the city (25.3% vs. 13.8% among RHWs, p = 0.02; 83.6% vs. 58.6% among CSWs, p = <0.01). Adjusting for other factors, “tried female cigarette brands” had the strongest association with current smoking (OR 5.69, 95%CI 3.44 to 9.41) among participants who had ever tried smoking.

**Conclusions/Significance:**

Exposure to female cigarette brands may increase the susceptibility to smoking among rural-to-urban migrant women. Smoke-free policies and increased taxes may be effective in preventing rural-to-urban migrant women from smoking initiation.

## Introduction

The tobacco epidemic in China is responsible for an enormous burden of disease and poses a significant challenge to the country's public health system [1]. China ratified the Framework Convention on Tobacco Control (FCTC) in 2005 and has begun to implement a number of tobacco control measures [2]. However, due to low prevalence of smoking among women; little attention has been given to tobacco prevention in this population. According to the nationally representative Global Adult Tobacco Survey conducted in China in 2010, the estimated prevalence of current smoking was 2.4% among women, which corresponds to about 15 million female smokers in China [3]. However, the national tobacco survey results in 1996 and 2002 show the proportion of young females smoking increasing, especially among women aged 15–19 [4], [5]. While male smoking rates have peaked and are in slow decline, female smoking rates are still rising [6]. There is a need for ongoing monitoring and research on tobacco use among women in China, particularly among vulnerable segments of the population, such as rural women who migrate to urban centers for employment.

In 2009, there were approximately 211 million rural-to-urban migrants in China and 49.6% were women [7]. These women may be vulnerable to risky health behavior as they are newly exposed to risk factors in the urban environment. In recent years, the social and economic environment in Chinese cities has been rapidly changing, and potentially associated with increased susceptibility to smoking among young women [8]. For example, changing gender norms may have significantly weakened the previously widespread social stigma against smokers among women. A recent qualitative study of female high school students suggests that smoking among women has become increasingly socially acceptable [9]. A typical response among participants was “smoking is quite normal—if men can smoke, then why can't women?” Historically, trans-national tobacco companies have exploited social change and increasing affinity to Western culture in low and middle income countries to expand the market for cigarette products among youth and women [10]. In fact, a study of multi-national tobacco company documents revealed plans to target Chinese women as a major, previously-untapped market for expansion of tobacco products [11]–[13]. Tobacco industry marketing efforts targeting young women include the introduction of candy-flavored cigarettes and women-specific brands [11]–[13]. A recent study has shown that exposure to women-specific cigarette brands is associated with a higher prevalence of smoking among young Chinese women [14].

For rural-to-urban migrants, navigating life in the urban environment is often a source of psychosocial stress which, in turn, may lead to increased tobacco use as a coping mechanism. Poor living conditions, unstable employment, social stigma, discrimination, and lack of social support also contribute to stress and high rates of depression [15]. There may also be significant social pressure for rural-to-urban migrant women to conform to the perception of “modern” women as modeled by other urban women. However, little research has explored the association between these factors and smoking among rural-to-urban migrant women in China.

In 2008, we conducted a survey of rural-to-urban migrant women in Beijing and found current smoking prevalence at 6.5% among hotel and restaurant workers (RHWs) and at 33.3% among commercial sex workers (CSWs) [16]. We also found that exposure to tobacco marketing in urban areas was associated with increased prevalence of smoking among rural-to-urban migrant women. However, the study was limited by a small sample size. Furthermore, since the study was conducted at only one site, it is unknown whether the findings are generalizable to the greater population of rural-to-urban migrant women in China. The objective of the present study was to describe factors associated with smoking and experimentation with smoking after migration among young rural-to-urban migrant women working in the hotel/restaurant and commercial sex industries in China. We focused on young women since they are more likely to accurately recall factors associated with smoking experimentation and initiation which typically occur during adolescence [17].

## Methods

### Study population

We conducted a cross-sectional survey of rural-to-urban migrant women in ten provincial capital cities in China. The study sites were selected based on previous estimates of smoking prevalence among women to ensure that provinces representing varying levels of smoking among women are included in our study. First, two provinces were selected from each of the four levels of smoking prevalence among women as reported in the 2002 Behavior Risk Factor Surveillance System (0.31%–1.18%, 1.18%–2.37%, 2.37%–4.71%, 4.71%–17.32%) [5]. We also selected two provinces with no data available in the 2002 survey. Final study sites were provincial capitals of Chongqing, Hohhot, Guangzhou, Nanchang, Kunming, Lanzhou, Hefei, Shanghai, Beijing and Shenyang, which represent major urban areas with large rural-to-urban migrant populations.

We prioritized RHWs and CSWs because established mechanisms exist that allowed for systematic sampling and recruitment of these two groups of rural-to-urban migrant women. For RHWs, we enrolled a consecutive convenience sample of approximately 150 rural-to-urban migrant women from district-level medical examination facilities at each study site. By law, service workers are mandated to undergo annual medical examinations at an approved facility. Study participants were recruited from 77 district-level facilities at the10 study sites, which provide medical examinations to 650,000 workers per year. The number of participants enrolled from each facility was determined based on the estimated proportion of rural-to-urban workers served at each facility from Aug. to Oct. in 2009 for the site.

For CSWs, approximately 50 women were enrolled from bars, night clubs, and karaoke parlors at each provincial site. The China Centers for Disease Control (CCDC) investigators at study sites have maintained relationships with managers of local entertainment venues where they routinely provide health education and conduct HIV prevention research. Permission from venue managers was obtained by CCDC investigators prior to each recruitment visit. A convenience sample of potential participants was approached and offered enrollment into the study.

Eligible participants were: (1) women with rural *hukou (a permanent residential record)*; (2) 18–24 years of age for women who were employed as a service worker in a restaurant/hotel (or unemployed but previous employment was in the restaurant/hotel industry); or (3) CSWs working in entertainment venues aged 18–30 years old. For CSWs, the upper limit for eligible age was increased to 30 years because CSWs tend to be older and enrolling a sufficient number of CSWs at a younger age range would not have been feasible within the study period.

### Study instrument

The survey instrument was developed based on existing survey items from the China National Tobacco Survey (1996) [4], China Behavior Risk Factor Surveillance System (BRFSS) Survey (2002) [5], China Global Adult Tobacco Survey (GATS) (2010) [3], and our previous study questionnaire–Susceptibility to Smoking Initiation among Rural and Urban Young Chinese Women (2007) [14]. All survey items were developed in English, translated into Chinese, and then back-translated into English by bilingual investigators who had experience in tobacco control. The questionnaire was pretested in Beijing and Inner Mongolia Autonomous Region prior to study initiation, and was modified based on pretest feedback. The survey collected the following information: (1) socioeconomic and demographic data; (2) general health behaviors; (3) smoking related behaviors; (4) smoking environment and attitudes, including attitudes towards female smoking; (5) smoking and anti-smoking advertisement and marketing, including exposure to female brands and product promotion activities; (6) knowledge of health risks and attitudes towards anti-smoking policies.

### Data collection

We conducted face-to-face interviews using handheld computers for electronic data capture. The questionnaire was designed using Questionnaire Development System 2.6 (Nova Research Company, Bethesda, Maryland, USA). Before the field study, we conducted three days of training for all site staff, which included two interviewers and one coordinator per province. At each study location (medical facility or entertainment venue), a private interview area was arranged to protect the participant's confidentiality during the interview. Study interviewers administered the anonymous survey after obtaining verbal informed consents from eligible participants. The survey was designed to take 20 minutes to complete. A small gift was provided to participants as a gesture of thanks according to Chinese custom. The Beijing-based study coordinator supervised survey administration via email and telephone, and provided consultation with the local CDC interviewers. In addition,provincial supervisors observed 10% of the interviews to ensure the quality of the data collection process for each interviewer. Field work was completed between July and November, 2010.

### Institutional review

The protocol used in this study received approval from the Institutional Review Boards at Peking Union Medical College and San Diego State University. Study interviewers administered the anonymous survey after obtaining verbal informed consents from eligible participants. Verbal permissions were recorded by the interviewers on the intake screens of the hand-held data collection devices. Because we were testing the use of hand-held electronic data collection methods in this survey, we did not utilize paper records or hard copy surveys. Individuals could and did refuse to participate, and all records of this refusal or agreement were recorded on the electronic devices and transferred to the password protected data files in the data warehouse. These files reside on a laptop, which served as the data warehouse for the QDS software. Records of verbal agreement to participate are accessible in the main data files. The ethics committees at both SDSU and PUMC approved this specific verbal permission process, as it was integral to the testing of the hand held, non-paper data collection scheme used in this research.

### Data analysis

Participants were categorized as “ever tried smoking” if they answered yes to “Have you ever smoked cigarettes, even if just one or two cigarette puffs?” Participants were further categorized as having first tried smoking prior to or after migration to the city. Current smokers were defined as participants who answered, “I have smoked everyday in the past 30 days” or “I have smoked in the past 30 days but have not smoked everyday” in response to “During the past 30 days, have you smoked cigarettes?”

Susceptibility to smoking was measured using the four following questions, “Do you think that you will smoke a cigarette soon? “, “Do you think you will smoke a cigarette in the next year?”, “Do you think that in the future you might experiment with cigarettes?”, and “If one of your best friends were to offer you a cigarette, would you smoke it?” Those who did not answer “definitely not” to all four questions were considered as susceptible [17].

We computed frequencies (%) of the sample characteristics stratified by employment type (RHWs or CSWs). The χ^2^ test was performed to examine associations between smoking outcomes and potential correlates. We evaluated two smoking outcomes: 1) first tried smoking after migrating to work in cities (among participants who had not tried smoking before migration) and 2) current smoking (among participants who had ever tried smoking). The final multivariate logistic model included employment type, age, level of education, age at first migration to the city for work, monthly income, and variables that were associated with the smoking outcome with a significance level of 0.05 or lower. Adjusted odds ratios (OR) were calculated for the two outcomes. All statistical analyses were conducted using SAS 9.0 (SAS Institute, Cary, North Carolina, USA).

## Results

We enrolled 1697 RHWs and 532 CSWs representing a 98.7% participation rate of eligible rural-to-urban migrant women who were offered enrollment. The non-response rates were 1.7% and 0.2% for RHWs and CSWs, respectively. The demographic characteristics of the study population are presented in Table 1. Study participants were mostly of Han ethnicity, had completed at least middle school education, were single, and lived with others. Most study participants first migrated to work in cities at the age of 18 to 19. Mean monthly income was 1229 China Yuan (CNY) for RHWs and 4221 CNY for CSWs (Table 1).

**Table 1:** Participant characteristics, restaurant/hotel workers (RHWs) and commercial sex workers (CSWs), China 2010.

Characteristic		RHWs(N = 1697)n (%)	CSWs(N = 532)n (%)
Age	Mean ± SD	20.6±1.8	23.1±3.3
Ethnicity	Han	1552(91.5)	487 (91.5)
	Minority	145 (8.5)	45 (8.5)
Education	Up to primary school completed	17(1.0)	22(4.1)
	Less than secondary school completed	73(4.3)	46(8.7)
	Secondary school completed	551(32.5)	183 (34.4)
	High school completed/technical secondary school	728 (42.9)	213 (40.0)
	College/university completed & higher	325(19.2)	68 (12.8)
	Don't know	3(0.2)	0 (0.0)
Marital status	Single	1421 (83.7)	360 (67.7)
	Married	195 (11.5)	113(21.2)
	Cohabitating	76 (4.5)	40(7.5)
	Divorced & Widowed	4 (0.2)	15 (2.8)
	Refuse to answer	1 (0.1)	4 (0.8)
Living situation	Live alone	144 (8.5)	79 (14.9)
	Live with others	1553 (91.5)	453 (85.2)
Age at first migration to city for work	Mean ± SD	18.41±2.14	18.83±3.05
Monthly income	Mean ± SD	1228.6±942.2	4220.8±3404
Smoking history	Ever tried smoking	312 (18.4)	310 (58.3)
	Ever tried female brand cigarettes	141(8.3)	234(44.0)
	Current smoker	54 (3.2)	223 (41.9)
	Susceptible to smoking among non current smokers	232 (15.3)	62 (23.4)
Smoking environment	Parents smoke	1084(63.9)	377(70.9)
	Friends smoke	902(53.2)	441(82.9)
	Manager of workplace smokes	299(17.6)	245(46.1)
	Customer smokes	990(58.3)	496(93.2)
	Boyfriends smokes	409(24.1)	278(52.3)

**Figure 1:**
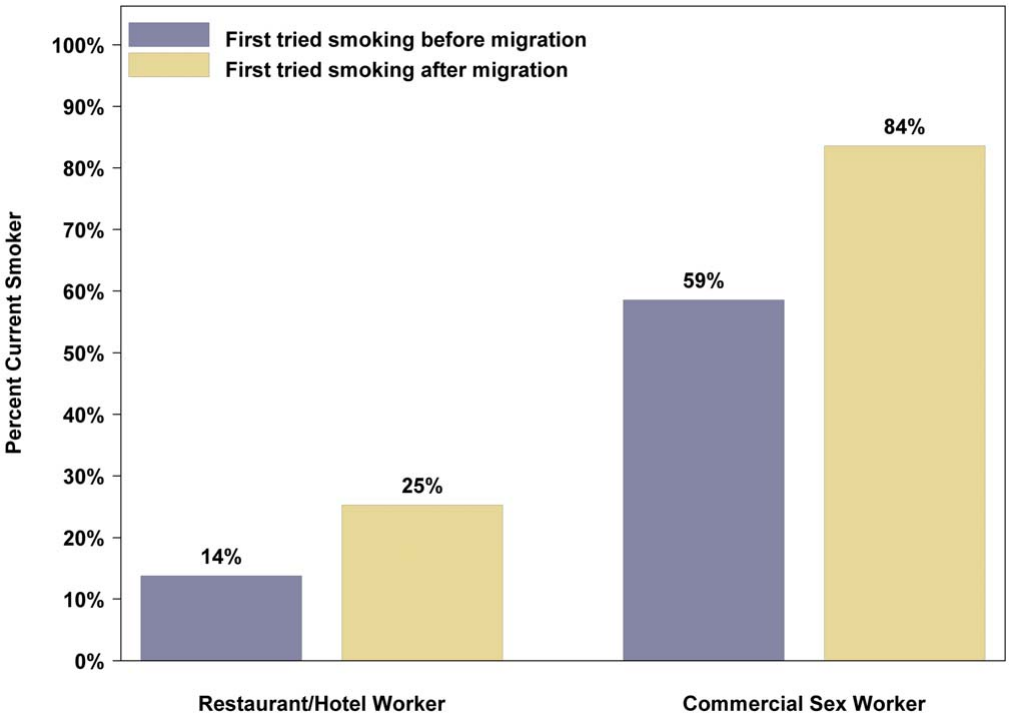
Prevalence of current smoking among those rural-to-urban migrant women have ever tried smoking, China 2010.

Overall, 312 (18.4%) RHWs and 310 (58.3%) CSWs had ever tried smoking. There were 54 (3.2%) and 223 (41.9%) current smokers among RHWs and CSWs, respectively. Participants who first tried smoking after moving to the city were more likely to be current smokers compared to participants who first tried smoking before moving to the city (25.3% vs. 13.8% among RHWs, p = 0.02; 83.6% vs. 58.6% among CSWs, p<0.01) (Figure 1). The rate of ever tried female brand cigarettes was higher for RHWs who first tried smoking after vs. before moving to the city (61.1% vs. 41.9%, p = <0.01), while the rates were similar among CSWs (83.0% vs. 80.7%, p = 0.60). In addition, more than 15% of the non-smokers were susceptible to smoking.

Among current smokers, nearly half of the RHWs reported their home, while half of the CSWs reported their workplace, as the location where they smoked most often, but most of them would support tobacco control policies that would prohibit smoking at work and at public places. The most common method of obtaining cigarettes during the past 30 days was purchasing cigarettes on their own, and the average percent of monthly income spent on tobacco was less than 2.5%. More than 60% of the participants reported that they would reduce cigarette consumption or quit if cigarette prices increased. Among daily smokers, 25.0% RHWs and 56.2% CSWs reported smoking within 30 minutes after waking up in the morning (Table 2).

**Table 2:** Characteristics of current smokers, restaurant/hotel workers (RHWs) and commercial sex workers (CSWs), China 2010.

Characteristic		RHWs(N = 54)n (%)	CSWs(N = 223)n (%)
Past 30 days place smoked most often	At home	24(44.44)	73 (32.74)
	At a friend or workmates home	5 (9.26)	15 (6.73)
	Inside work	2 (3.70)	109 (48.88)
	Outside	15 (27.78)	17 (7.62)
	Another location	8 (14.81)	9 (4.04)
Past 30 days most common method cig obtained	Self-purchased	28 (51.85)	137 (61.43)
	Obtained from workmate or friends	24 (44.44)	76 (34.08)
	Other	2 (3.70)	10 (4.48)
Expenditure on tobacco as a percentage of monthly income monthly (%)		2.0	2.5
Self-reported effect of increase in cigarette prices	Reduce or quit smoking	37 (68.52)	142 (63.68)
	No effect	17 (31.48)	81 (36.32)
Knowledge and attitude towards China anti-tobacco policies	Support prohibiting smoking at work	38(70.37)	150(67.26)
	Support prohibiting smoking at public places	33(61.11)	154(69.06)
Who provided you with the cigarette when you tried your first cigarette puff?	Took somebody else's cigarette	10(18.52)	45(20.18)
	Bought cigarette myself	6(11.11)	44(19.73)
	Workmate/school mate/friend gave it to me	37(68.52)	122(54.71)
	Other	1(1.85)	12(5.38)

The results of the multivariate analysis of the correlates of a) first tried smoking after coming to the city among participants who had never tried smoking prior to moving to the city and b) current smoker among participants who had ever tried smoking are shown in Table 3. Factors independently associated with greater odds of having tried smoking in the city were older age, commercial sex work, and having friends, a manager, or a boyfriend who smoke. Higher education level, older age at migration and supporting prohibition of smoking at workplace were negatively associated with having first tried smoking after migration. Among participants who had ever tried smoking, factors independently associated with higher odds of current smoking included commercial sex work, having a manager who smokes, having a boyfriend who smokes, and having tried female brand cigarettes. Monthly income and supporting prohibition of smoking at public places were negatively associated with current smoking.

**Table 3:** Correlates of having first tried smoking after coming to the city and current smoking^a^.

Correlates	First tried smoking in the city^b^ OR (95% CI)	Current smoker^c^ OR (95% CI)
Type of worker		
RHW	1.00	1.00
CSW	4.28 (2.88 to 6.35)	11.98 (6.68 to 21.47)
Current age	1.10 (1.03 to 1.18)	1.05 (0.96 to 1.14)
Level of education		
Less than secondary school	1.00	1.00
Secondary/High school/technical	0.47 (0.28 to 0.76)	0.52 (0.26 to 1.04)
College/university & higher	0.43 (0.21 to 0.85)	0.52 (0.21 to 1.31)
Age at first migration to city for work	0.81 (0.75 to 0.86)	0.99 (0.91 to 1.09)
Monthly income		
< = 1000CNY	1.00	1.00
>1000CNY	1.21 (0.79 to 1.86)	0.32 (0.17 to 0.60)
Friends smoking		
No	1.00	1.00
Yes	1.96 (1.26 to 3.04)	1.54 (0.79 to 3.01)
Manager smokes		
No	1.00	1.00
Yes	1.62 (1.14 to 2.30)	2.19 (1.35 to 3.53)
Boyfriends smoking		
No	1.00	1.00
Yes	2.21 (1.56 to 3.13)	1.92 (1.21 to 3.05)
Tried female cigarette brands		
No	——	1.00
Yes	——	5.69 (3.44 to 9.41)
Support ban on smoking in the workplace		
Other	1.00	1.00
Agree	0.54 (0.31 to 0.92)	0.78 (0.38 to 1.60)
Support ban on smoking in public places		
Other	1.00	1.00
Agree	0.94 (0.57 to 1.56)	0.45 (0.22 to 0.92)

a Based on multiple logistic regression analysis.

b Among 1802 participants who had never tried smoking prior to migrating to the city.

c Among 594 participants who had ever tried smoking.

## Discussion

The prevalence of current smoking among restaurant/hotel workers and CSWs in our study was more than 5-fold and 60-fold higher, respectively, than the current nationally representative estimates of 0.7% reported among women aged 15–24 in the China GATS report [3]. The prevalence estimates among hotel and restaurant workers were comparable to estimates of 1.3% daily and 1.8% occasional smoking among rural-to-urban migrant women in three Chinese cities as reported by Yang and colleagues [18]. Chen and colleagues found a prevalence of 10.9% current smoking among rural-to-urban migrant women in Beijing which is higher than our estimate among restaurant and hotel workers but lower than our estimate among CSWs [19]. These differences may be due to the inclusion of women from more diverse occupational settings in their study. Our study has shown that the prevalence of smoking differed substantially between the occupational groups included in our study population.

Findings from our study suggest that the social and work environment that rural-to-urban migrant women encounter in the city is associated with smoking initiation. Many rural-to-urban migrant women smokers reported obtaining their first cigarette from their workmate or friend. In addition, CSWs who currently smoke were likely to report smoking inside their workplace (entertainment venue). Policies that mandate smoke-free workplaces are an important component of the FCTC and have been shown to be effective in reducing smoking rates in other settings [2], [20]. In China, smoke-free policies also have been introduced in public transportation, universities and hospitals [2]. However, there have been limited efforts to establish smoke-free policies in hotels, restaurants, and entertainment venues. In our study, most participants supported workplace smoking bans. Such policies may be effective in preventing smoking initiation among women who work in these industries, in addition to reducing their exposure to second-hand smoke.

Exposure to cigarette brands made for women was also associated with smoking among rural-to-urban migrant women. Among women who have ever tried smoking, the prevalence of current smoking among women who had tried female brand cigarettes was substantially higher than that among women who had not tried female brands. Such branding and packaging methods have been shown to be highly effective in introducing cigarettes to young women in other settings [10]. To protect girls and young women from such marketing practices, tobacco control advocates should explore regulations to restrict the packaging and advertisement for female targeted cigarettes brands. Furthermore, China has yet to implement full recommendations for evidence-based warning labels on cigarette packaging [2]. On April 2, 2008, the State Tobacco Monopoly Administration and the General Administration of Quality Supervision, Inspection and Quarantine, jointly released the Regulations on Cigarette Packaging and Labeling in the Territories of the People's Republic of China (the Labeling Regulations) [21]. However, the new cigarette package design mandated by the regulation did not comply with FCTC requirements, leading to inadequate health warnings on cigarette packages [22]. Additional restrictions and requirements for packaging and advertisement should be implemented and existing bans should be carefully monitored and enforced to ensure that the tobacco epidemic is prevented among young women in China.

Among study participants, the percentage of monthly income spent on tobacco was low, and a majority of the participants reported that they would reduce cigarette consumption or quit if the price of cigarettes increased. In 2009, the Chinese government announced an increase in tobacco taxes. However, the policy did not result in increase in the retail price of a pack of cigarettes [23]. Increasing the retail price of cigarettes by imposing taxes has been shown to be effective in reducing smoking, particularly among young people and people with low income [24]. Smokers with lower dependency on nicotine are also more likely to quit or reduce smoking as a response to increase in cigarette prices [24]. In our study, restaurant and hotel workers reported very low levels of income and most current smokers reported low levels of nicotine dependence. Therefore, tax policies that result in an increase in the price of cigarettes may be particularly effective in reducing smoking levels and preventing smoking initiation in this population.

Our study had several limitations. First, due to the cross-sectional design employed in the study, it is impossible to assert a causal relationship between potential risk factors and smoking behavior. Prospective studies are needed to better characterize the relationship between the factors associated with smoking and smoking behavior among rural-to-urban migrant women. In addition, current smoking status was defined based on self-report only, which has been shown to underestimate smoking prevalence in certain settings [25]. We sought to provide a safe, private environment for interviews to reduce the likelihood of under-ascertainment. Biochemical confirmation should be used to obtain more precise estimates of smoking prevalence. Finally, the study results represent rural-to-urban migrant women working in restaurant and hotel industries and commercial sex work in ten provincial capitals. Our findings may not be generalizable to rural-to-urban migrant women employed in other occupations, nor are they representative of national rates.

We present results from a large study specifically designed to investigate factors related to smoking among rural-to-urban migrant women in China. The tobacco epidemic already appears to be firmly established among rural-to-urban migrant women who are CSWs and may emerge among restaurant and hotel workers. The findings from our study have direct policy implications and should contribute to the design of evidence-based measures to prevent smoking among rural-to-urban migrant women in China. Specifically, smoke-free policies and increased taxes may be effective in preventing rural-to-urban migrant women from smoking initiation. Additional research is needed to evaluate the effectiveness of tobacco control interventions in this population.
